# 

**Published:** 1843-04-29

**Authors:** 


### CONTENTS.

A Course of Clinical Lectures, delivered at the Hôtel Dieu, Paris, for the Session 1842–'43. By A. F. Chomel, M.D. Lecture 6. Scirrhous Degeneration of the Liver, . . . . . . . 85

*Philadelphia Hospital.* Dr. Wilson's Case of Phthisis Pulmonalis, . . . . 86

*Bibliographical Notices.*

The Elements of Materia Medica and Therapeutics. By Jonathan Pereira, M.D., F.R.S. and L.S. &c. With Numerous Notes and Illustrations. From the Second London Edition, Enlarged and Improved. With Notes and Additions. By Joseph Carson, M.D. 2 vols. 8vo. . . . . 87

Transactions of the Medical Society of the State of New York, . . . . 88

Operations for Fissure of the Soft and Hard Palate. By J. Mason Warren, M.D. . . . . . . . . 88

*Foreign Correspondence,* . . . . 89

Editorial—Medical Officers of the Navy, . . . . . . . . 91

*Retrospect of the Medical Sciences.*

Employment of Muriate of Ammonia in Scirrhus of the Stomach, . . . . 94

Structure of the Arteries, . . . . 94

Influence of improved Diet on the Health of the Insane, . . . . . . 95

On Fatty Degeneration of the Arteries, . 95

A Normal and Abnormal Conscious State, 95

Carbonic Acid formed in Typhus, . . 95

Introduction of Air in the Veins, . . 95

Electro-Puncture, . . . . . . . 96

Renal Disease simulating Calculus in the Bladder, . . . . . . . 96

Observations on Impulsive Insanity, . 96

